# Human salivary histatin‐1 (Hst1) promotes bone morphogenetic protein 2 (BMP2)‐induced osteogenesis and angiogenesis

**DOI:** 10.1002/2211-5463.12906

**Published:** 2020-06-29

**Authors:** Ping Sun, Andi Shi, Chenxi Shen, Yi Liu, Gang Wu, Jianying Feng

**Affiliations:** ^1^ The Affiliated Stomatology Hospital Zhejiang University School of Medicine Hangzhou China; ^2^ Key Laboratory of Oral Biomedical Research of Zhejiang Province Zhejiang University School of Stomatology Hangzhou China; ^3^ Department of Oral and Maxillofacial Surgery/Pathology Amsterdam UMC and Academic Center for Dentistry Amsterdam (ACTA) Vrije Universiteit Amsterdam (VU), Amsterdam Movement Science (AMS) Amsterdam the Netherlands; ^4^ Laboratory for Myology Department of Human Movement Sciences Faculty of Behavioural and Movement Sciences Vrije Universiteit Amsterdam (VU), Amsterdam Movement Sciences (AMS) Amsterdam the Netherlands; ^5^ Key Laboratory of Oral Medicine Guangzhou Institute of Oral Disease Affiliated Stomatology Hospital of Guangzhou Medical University Guangzhou Medical University Guangzhou China; ^6^ Department of Oral Implantology and Prosthetic Dentistry Academic Centre for Dentistry Amsterdam University of Amsterdam (UvA) and Vrije Universiteit Amsterdam (VU) Amsterdam the Netherlands; ^7^ School of Dentistry Zhejiang Chinese Medical University Hangzhou China

**Keywords:** angiogenesis, bone morphogenetic protein 2, bone regeneration, histatin‐1, osteogenesis, peptide

## Abstract

Large‐volume bone defects can result from congenital malformation, trauma, infection, inflammation and cancer. At present, it remains challenging to treat these bone defects with clinically available interventions. Allografts, xenografts and most synthetic materials have no intrinsic osteoinductivity, and so an alternative approach is to functionalize the biomaterial with osteoinductive agents, such as bone morphogenetic protein 2 (BMP2). Because it has been previously demonstrated that human salivary histatin‐1 (Hst1) promotes endothelial cell adhesion, migration and angiogenesis, we examine here whether Hst1 can promote BMP2‐induced bone regeneration. Rats were given subcutaneous implants of absorbable collagen sponge membranes seeded with 0, 50, 200 or 500 μg Hst1 per sample and 0 or 2 μg BMP2 per sample. At 18 days postsurgery, rats were sacrificed, and implanted regional tissue was removed for micro computed tomography (microCT) analyses of new bone (bone volume, trabecular number and trabecular separation). Four samples per group were decalcified and subjected to immunohistochemical staining to analyze osteogenic and angiogenic markers. We observed that Hst1 increased BMP2‐induced new bone formation in a dose‐dependent manner. Co‐administration of 500 μg Hst1 and BMP2 resulted in the highest observed bone volume and trabecular number, the lowest trabecular separation and the highest expression of osteogenic markers and angiogenic markers. Our results suggest that coadministration of Hst1 may enhance BMP2‐induced osteogenesis and angiogenesis, and thus may have potential for development into a treatment for large‐volume bone defects.

## Graphical abstract

Sufficient bone tissue is important for aesthetics and musculoskeletal functions. We showed that coadministration of human salivary histatin‐1 enhanced new bone formation (shown by microCT analysis) and angiogenesis (indicated by immunostaining of CD105, as shown by the black arrows) induced by bone morphogenetic protein 2 in a classic ectopic bone induction model using absorbable collagen sponge.

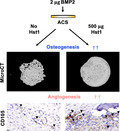

**Abbreviations**
ACS: absorbable collagen sponge
ALP: alkaline phosphatase
BMP2: bone morphogenetic protein 2
B.S.: bone surface
BV: bone volume
FGF: fibroblast growth factor
G: group
Hst1: histatin‐1
IHC: histoimmunochemistry
MSC: mesenchymal stem cell
n.s.: not significant
SD: Sprague Dawley
Tb.N.: trabecular number
Tb.Pf.: trabecular pattern factor
Tb.Sp.: trabecular separation
Tb.Th.: trabecular thickness
VEGF: vascular endothelial growth factor

In the fields of periodontology, implantology and maxillofacial surgery, large‐volume bone defects can result from congenital malformation, trauma, infection, inflammation and cancer. Hitherto, it is still highly challenging to completely repair these bone defects with current clinically available interventions [1]. Autografts are associated with severe donor‐site pain and morbidity. Allografts, xenograft and most synthetic materials have no intrinsic osteoinductivity [2, 3], a property to induce the osteogenic differentiation of pluripotent mesenchymal stem cells (MSCs) and the formation of bone at a nonosseous site, so that they cannot facilitate complete bone repair of large defects when applied alone. One common approach in clinic is to coadministrate particulate autografts to supply the necessary osteogenic elements, such as growth factors and osteogenic cells [1]. However, in this case, the limitations of autografts cannot be avoided.

An efficient alternative approach is to functionalize the biomaterial with osteoinductive agents, such as bone morphogenetic protein 2 (BMP2) [2, 4, 5]. BMPs belong to the transforming growth factor superfamily, and many BMP members, such as BMP2, BMP4, BMP6, BMP7 and BMP9, exhibit strong osteoinductivity to induce *de novo* bone formation in nonosseous sites [6]. In particular, BMP2 and BMP7 in combination with absorbable collagen sponge (ACS) membrane have been approved by the US Food and Drug Administration to repair various bone defects and to facilitate spine fusion [6, 7]. The osteoinductive effect of BMPs is initiated by their binding to two types of BMP receptors and forming a receptor complex, resulting in enhanced levels of phosphorylated Smad1/5 (p‐Smad1/5). p‐Smad1/5 forms a complex with Smad4 and translocates into the nucleus to induce the expression of osteogenic genes, leading to osteoblastogenesis and, finally, osteogenesis. Osteoblastogenesis and osteogenesis are symbolized in a series of biological events, such as alkaline phosphatase (ALP) expression (early differentiation marker), osteocalcin expression (late differentiation marker) and ossification [8, 9].

In contrast, there is still increasing demand in more rapid bone regeneration to facilitate earlier bone functionality and patient aesthetics. One of the hot topics to promote bone regeneration is to coadministrate bioactive agents to promote BMP2‐induced bone regeneration through various mechanisms [10]. For example, we recently proved that angiogenic agents, such as hyaluronic acid, promote BMP2‐induced bone regeneration because angiogenesis is a prerequisite for new bone regeneration [10]. Other angiogenic growth factors, such as fibroblast growth factors (FGFs) [11, 12] and vascular endothelial growth factor (VEGF) [13, 14], are also adopted to promote bone regeneration [15]. Apart from angiogenesis, approaches to promote cell migration and improve cell–scaffold interaction are also highly important for bone regeneration [16]. With this inspiration, we wish to adopt a bioactive agent that bears both above‐mentioned functions to significantly promote BMP2‐induced bone regeneration. One of such agents is human salivary histatin‐1 (Hst1), a member of a large histidine‐rich peptide family present in human saliva. It has been previously demonstrated that Hst1 promotes cell adhesion, migration [17] on hydroxyapatite and sputtered titanium [18], and wound closure [19, 20, 21]. Furthermore, Hst1 also shows a strong ability to promote endothelial cell adhesion, migration and angiogenesis [22]. Consequently, Hst1 shows a potential to enhance BMP2‐induced bone regeneration. To this end, the recently described angiogenic factor, Hst1, was used to evaluate whether BMP2‐induced angiogenesis and osteogenesis were improved.

In this study, we adopted a classical ectopic bone induction model to test the dose‐dependent effect of Hst1 on BMP2‐induced new bone formation. Our data showed that Hst1 significantly promoted BMP2‐induced osteogenesis in a dosage‐dependent way with simultaneously enhanced angiogenesis *in vivo*.

## Materials and methods

### Peptide synthesis and purification

The lyophilized linear Hst1 peptide with a purity of 95% was purchased from Hangzhou Huibo Science and Technology Company. Histatin stock solution was prepared by dissolving the lyophilized peptide in PBS in a concentration of 25 μg·μL^−1^ and stored at −20 °C until further use. The peptide solution was further diluted using PBS.

### Sample preparation and group setup

ACS (Medtronic Sofamor Danek, Memphis, TN, USA) membranes were cut into identically sized circular samples (5‐mm diameter). Samples with 50, 200 and 500 μg per sample were prepared by adsorbing 20 µL of 2.5, 10 and 25 μg·μL^−1^ Hst1 solution onto ACS discs, respectively. Two micrograms recombinant human BMP2 (Medtronic Sofamor Danek) per sample was also added on the ACS by adding 20 µL of 0.1 µg·µL^−1^ BMP2 stock solution. The dosage of BMP2 was determined as previously described [10]. The samples of these groups were prepared fresh before surgery; besides, samples were stored overnight under aseptic conditions in a sterile hood for induction of sample drying before implantation.

Experimental groups were set up as follows: group (G) 1 (G1), ACS + 20 µL sterile water; G2, ACS + 50 μg Hst1 per sample; G3, ACS + 200 μg Hst1 per sample; G4, ACS + 500 μg Hst1 per sample; G5: ACS + 2 µg BMP2; G6, ACS + 2 µg BMP2 + 50 μg Hst1 per sample; G7, ACS + 2 µg BMP2 + 200 μg Hst1 per sample; and G8, ACS + 2 µg BMP2 + 500 μg Hst1 per sample. The group information was kept blind to operators when they performed the animal surgery, microCT analysis and histological process. Animals were assigned randomly to the groups in which samples were implanted subcutaneously and symmetrically. Eight samples per group were implanted. Four samples were used for undecalcified tissue sectioning, and four samples were used for decalcified tissue sectioning.

### Animal surgery

The animal study was approved by the Ethical Committee of South Medical University, Guangzhou, China (No. L2018103). The animal experiment was carried out according to the ethics laws and regulation of South Medical University and adheres to the ARRIVE Guidelines for reporting animal research [23]. Eight‐week‐old specific pathogen‐free male Sprague Dawley (SD) rats (mean weight, 230 g; range, 190–250 g) were used in this study. The rats were accommodated for 1 week before surgery to get used to the new environment. The SD rats were kept in an animal experiment center (South Medical University Laboratory Animal Research Center, Guangzhou, China). Temperature for keeping the SD rats was 20–23 °C, day/night light cycle time was 14/10 (h/h), humidity was 60–80%, and sterile complete feed (Anlimo, Nanjing, China) and filtered water were freely available.

For the induction of a general anesthesia, 1% pentobarbital was intraperitoneally injected. Aseptic techniques were used during the surgical procedures. The iliac crest was used as the landmark for determining the location of the skin incision, and a 25‐mm posterior longitudinal incision was made bilaterally, 5–10 mm laterally from the midline. ACSs were implanted with or without BMP2 into the subcutaneous space of the lumbar back. Right after implantation, the soft tissues were repositioned, and the wound was closed using standard nonresorbable suture materials. The wound was then disinfected with 10% povidone–iodine. Animals were kept at 23 °C ambient temperature conditions until awakened.

### MicroCT analysis

All rats were sacrificed 18 days after surgery by intramuscular injection of excess Sumianxin II. The implanted regional tissue was harvested and immediately stored in 10% neutral‐buffered formalin for fixation. The samples were then scanned by microCT (microCT80; Scanco Medical, Bassersdorf, Switzerland) at a resolution of 10 μm (80 kV, 100 µA) and then subjected to offline reconstruction (gray value: 0–0.075). The three‐dimensional reconstructions and measurement were acquired to evaluate the bone volume (BV), trabecular thickness (Tb.Th.), trabecular separation (Tb.Sp.), trabecular number (Tb.N.), trabecular pattern factor (Tb.Pf.) and bone surface (B.S.).

### Undecalcified sectioning

The fixed specimens were rinsed in tap water, dehydrated in a graded EtOH series, embedded in methyl methacrylate and sectioned with a Leica microtome using a random sampling protocol as previously described [2, 10]. The sections were then mounted on a plexiglass board and stained by our well‐established McNeal’s Tetrachrome staining protocol. Under the light microscope, the newly formed bones were dark red, the nuclei were blue stained, the collagen fibers were pink and the soft tissue was blue. Sections were photographed at a final magnification of 2.5× under Zeiss light microscope (SteREO Discovery.V8; Zeiss, Oberkochen, Germany) and 200× under Leica light microscope (DM2500 & DM2500 LED; Leica, Wetzlarm, Germany). The reference (subcapsular) area and new bone area were histomorphometrically evaluated using a systematic random‐sampling protocol [24] and point counting method [25] as previously described [2, 3, 10]. The new bone percentage was calculated through dividing the total new bone area by the total subcapsular area.

### Decalcified tissue slicing

After being decalcified in 10% EDTA at 4 °C for 2–4 weeks, the specimens were dehydrated with alcohol and embedded in paraffin for immunohistochemical analysis. Four‐micrometer‐thick sections were deparaffinized in 100% xylene for 10 min and then hydrated with 100% EtOH for 5 min, 95% for 3 min, 80% for 3 min and 70% for 3 min. After being washed with distilled water, the sections were trypsin digested for 8 min for antigen retrieval, then rinsed in PBS for 5 min at least three times. Afterward, the slides were incubated with 3% hydrogen peroxide for 10 min and rinsed in PBS 5 min three times. Then the slides were incubated with primary antibodies for Runx2 (rabbit polyclonal antibody, ab192256; 1 : 600 dilution; Abcam, Cambridge, UK), collagen I (rabbit polyclonal antibody, ab21287; 1 : 400 dilution; Abcam, Cambridge, UK), ALP (rabbit polyclonal antibody, ab95462; 1 : 200 dilution; Abcam, Cambridge, UK), VEGF (rabbit polyclonal antibody, Ag13500; 1 : 200 dilution; Protein Tech, Wuhan, Hubei, China), FGF2 (mouse polyclonal antibody, SC‐365106 (G2), 1 : 150 dilution; Santa Cruz Biotechnology, Dallas, Texas, USA), CD105 (mouse polyclonal antibody; ab11414, 1 : 150 dilution; Abcam, Cambridge, UK), CD31 (rabbit polyclonal antibody, ab182981; 1 : 150 dilution; Abcam, Cambridge, UK) for 1 h in a humid chamber at 37 °C and shaken every 15 min. After rinsing in PBS, the slides were incubated with goat anti‐rabbit or mouse secondary antibodies labeled with horseradish peroxidase (ab6721; Abcam) for 30 min at 37 °C. Finally, the slides were incubated with 2,4‐diaminobutyric acid chromogen and examined for color change under light microscope (BX51; Olympus Japan Inc., Tokyo, Japan). Integrated optical density, positive cell number or positive cell rate were assessed under a light microscope using a computer‐based image analysis system (Image Pro Plus 6.0; Media Cybernetic, Silver Springs, MD, USA). Four randomly selected sections from the serial sections collected from each sample were analyzed manually.

### Statistical analysis

All data were presented as mean ± SD. Statistical analysis was performed by one‐way ANOVA using spss 13.0 software (SPSS, Inc., Chicago, IL, USA) followed by *post hoc* procedures based on Bonferroni’s test. For the data of histoimmunochemistry (IHC) analysis, we used two‐way ANOVA to analyze the data with Bonferroni’s test for multiple comparison. A *P*‐value <0.05 (two‐tailed) was considered statistically significant.

## Results

### MicroCT analysis

**Fig. 1:**
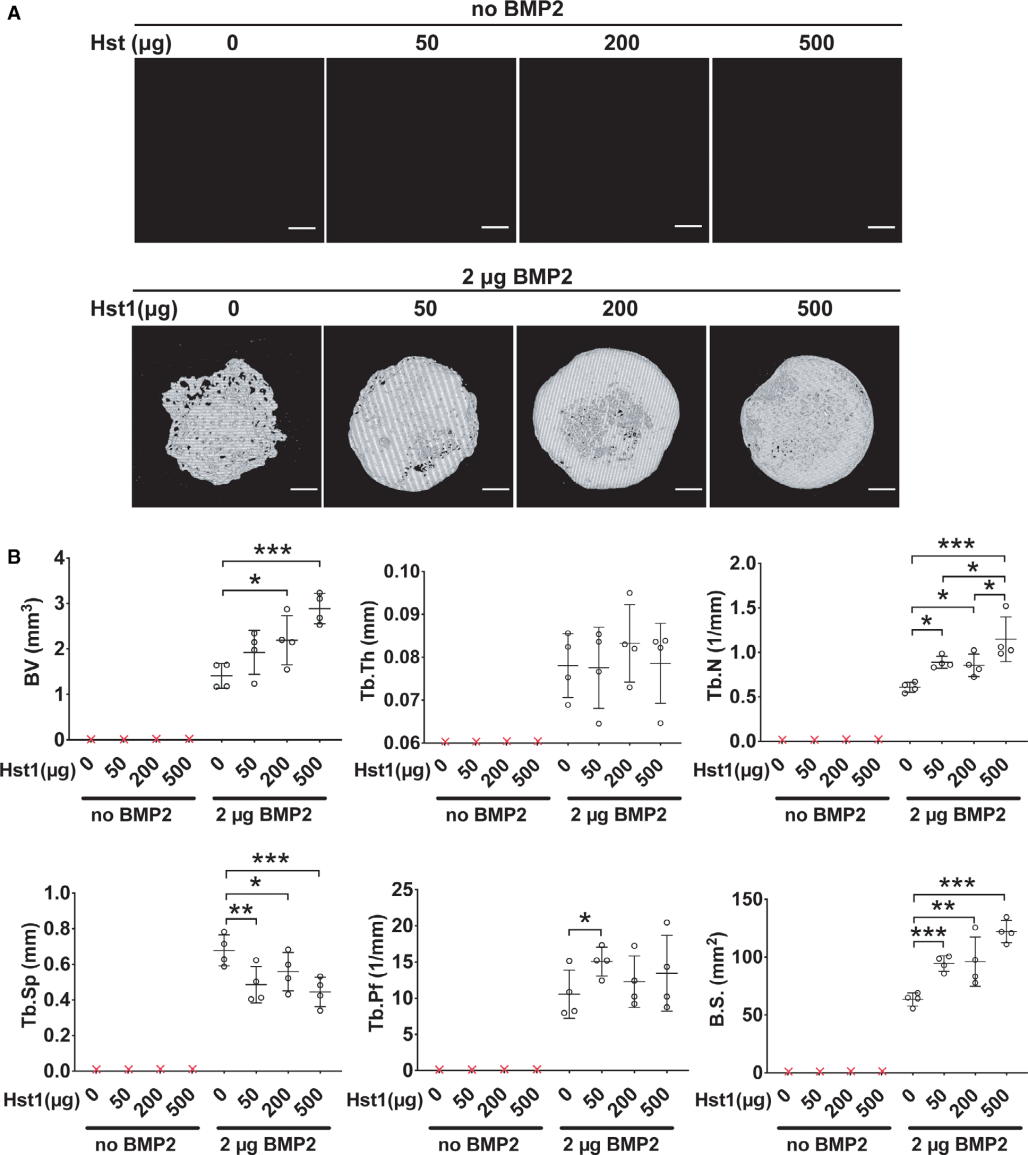
MicroCT analysis of newly formed bone on ACS membranes with 0, 50, 200 or 500 μg Hst1 per sample in the absence or presence of 2 μg BMP2 per sample that were implanted subcutaneously in rats for 18 days. (A) MicroCT images of Hst1/ACS constructs in the absence or presence of 2 μg BMP2 per sample. Scale bars: 1 mm. (B) Graphs depict the microCT analyses of the quantity and microstructure of newly formed bone on ACS membranes with 0, 50, 200 or 500 μg Hst1 per sample in the absence or presence of 2 μg BMP2 per sample. The following parameters were analyzed: BV, Tb.Th., Tb.N., Tb.Sp., Tb.Pf. and B.S. Data were presented as mean ± SD; *n* = 4 per group. Data were analyzed by one‐way ANOVA with Bonferroni’s test for multiple comparisons. Asterisks denote the level of statistical significance: *P < 0.05, **P < 0.01, ***P < 0.001.

MicroCT analysis showed that new bone formation was detected only in the four BMP2‐containing groups (Fig. 1A). Hst1 increased BMP2‐induced BV in a dose‐dependent manner, which was significant for 200 and 500 μg Hst1 per sample in comparison with the group of BMP2 alone. Five hundred micrograms Hst1 (2.8 ± 0.3 mm^3^) almost doubled the new bone formation in comparison with BMP2 alone (1.3 ± 0.2 mm^3^; *P* < 0.001). The addition of Hst1 could not significantly influence Tb.Th., but did significantly enhance Tb.N., with the highest effect occurring to 500 μg Hst1 (1.0 ± 0.2/mm). Consistently, the addition of Hst1 was also associated with significantly lower Tb.Sp., with the lowest value also occurring to 500 μg Hst1 (approximately reduced by 42% compared with the group of BMP2 alone; *P* < 0.001). The addition of Hst1 was associated with significantly higher Tb.Pf. at 50 μg per sample (13.5 ± 4.1/mm; *P* < 0.05). B.S. was significantly enhanced by Hst1 with almost doubled value at 500 μg (113 ± 15.9 mm^2^; *P* < 0.001; Fig. 1B).

### Histomorphometrical analysis

**Fig. 2:**
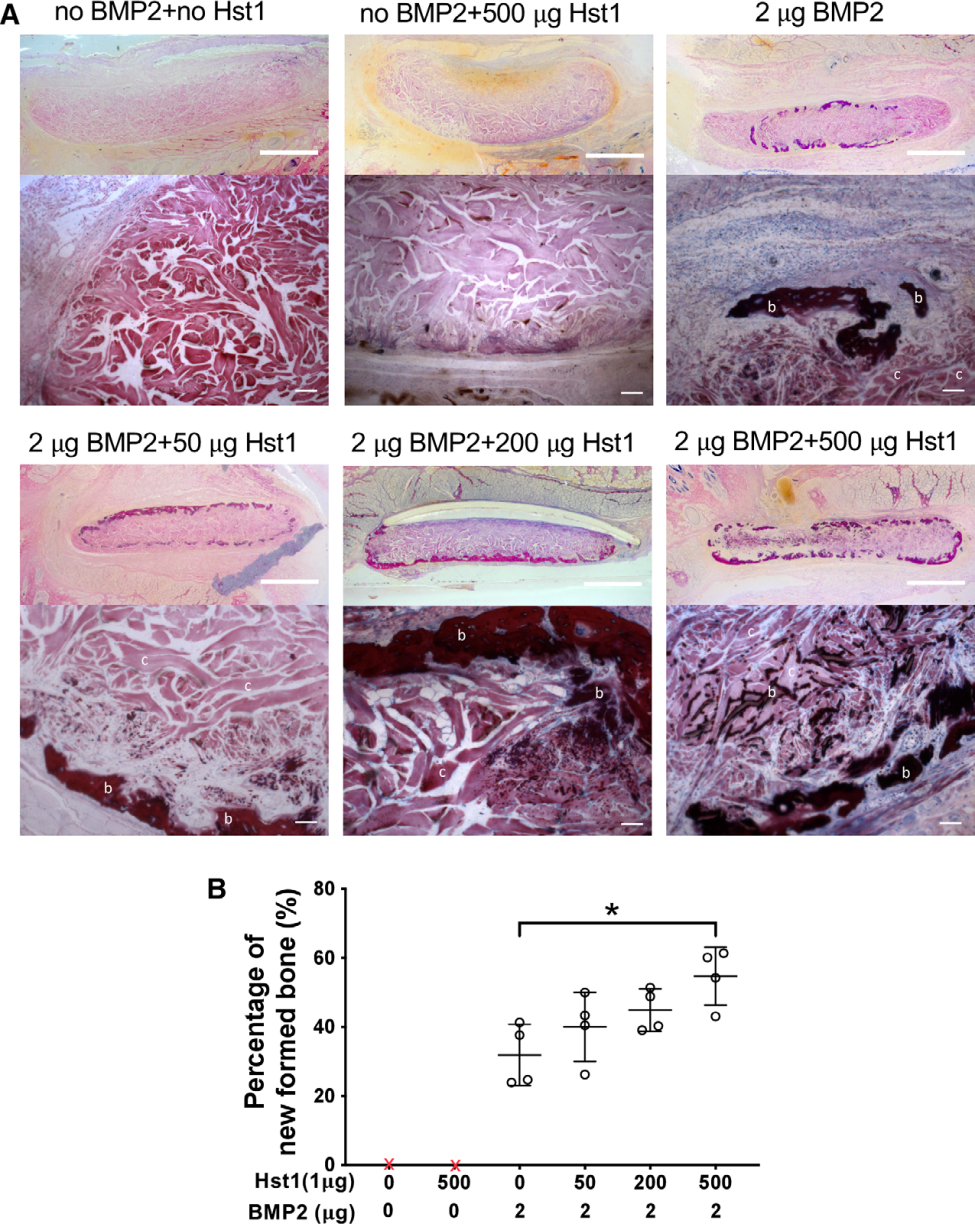
(A, B) Histological observations (A) and histomorphometrical analyses (B) of new bone formed on ACS membranes with 0, 50, 200 or 500 μg Hst1 per sample in the presence or absence of 2 μg BMP2 per sample. All samples were implanted subcutaneously in rats for 18 days. b, bone tissue; c, collagen fibers of ACS membrane. Scale bars: 1 mm in stereology images; 200 μm in high‐magnification images. Graph depicting area percentage of newly formed bone. Data were presented as mean ± SD; *n* = 4 per group. Data were analyzed by one‐way ANOVA with Bonferroni’s test for multiple comparisons. Asterisk denotes the level of statistical significance: *P < 0.05.

Histologically, in the group of BMP2 alone, new bone formed homogenously on the surface of ACS membranes; in contrast, the addition 500 μg Hst1 facilitated a homogeneous distribution of new bone both on the surface and within the ACS membrane. With the increase of Hst1 dosage, more calcified locations were found on ACS collagen fibers (Fig. 2A). The percentage of newly formed bone area to the total subcapsular area was significantly more in the group of BMP2 + 500 μg Hst1 than the BMP2 alone group (*P* < 0.05; Fig. 2B).

### IHC analysis of osteogenic markers

**Fig. 3:**
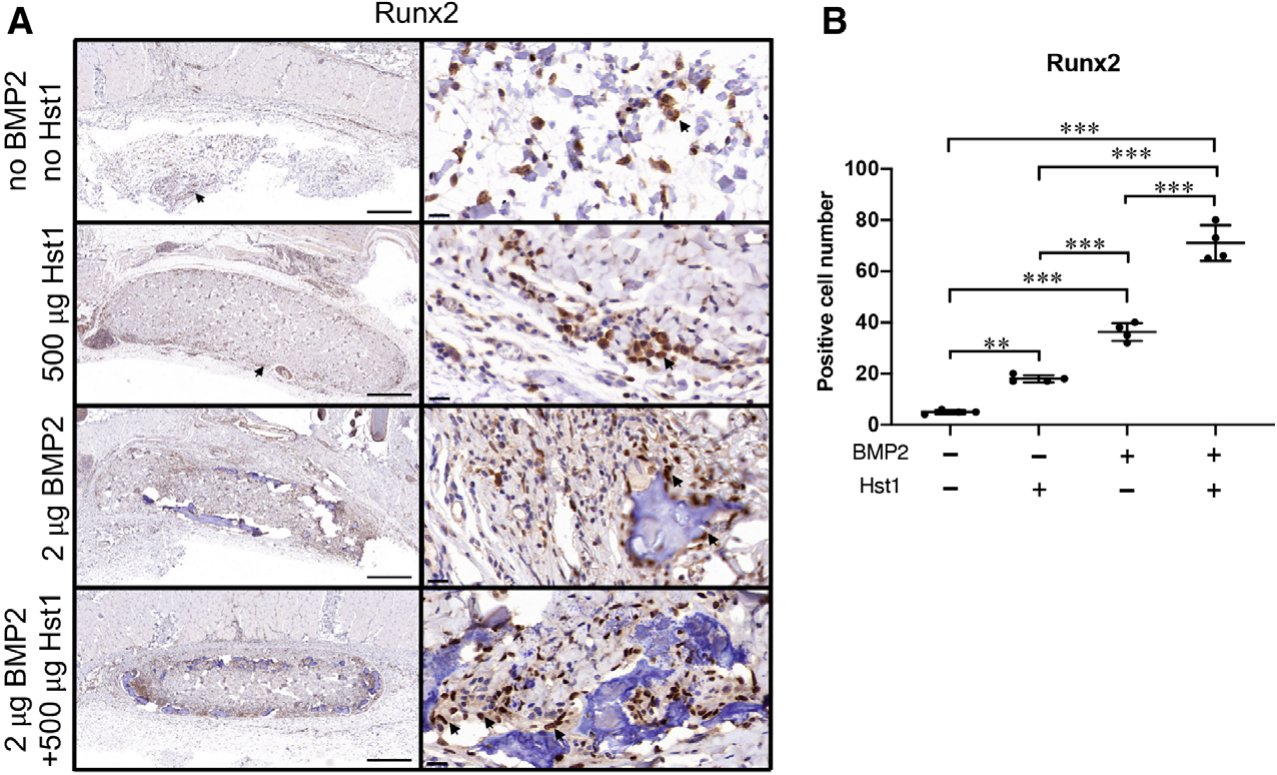
(A, B) Immunohistochemical micrographs (A) and quantitative analyses (B) of osteogenic marker Runx2 were performed on ACS in four groups: control group (no Hst1, no BMP2), 500 μg Hst1 per sample group, 2 μg BMP2 per sample group and 2 μg BMP2 per sample + 500 μg Hst1 per sample group. Samples were retrieved after an 18‐day subcutaneous implantation. Scale bars: 500 μm in lower‐magnification images; 20 μm in high‐magnification images. Cells with positive reaction were indicated using black arrows. Positive cell number within unit area (per 350.26 × 180.77 μm^2^) was quantified. Data were analyzed by two‐way ANOVA with Bonferroni’s test for multiple comparisons. Data are presented as mean ± SD; *n* = 4 per group. Asterisks denote the level of statistical significance: **P < 0.01, ***P < 0.001.

**Fig. 4:**
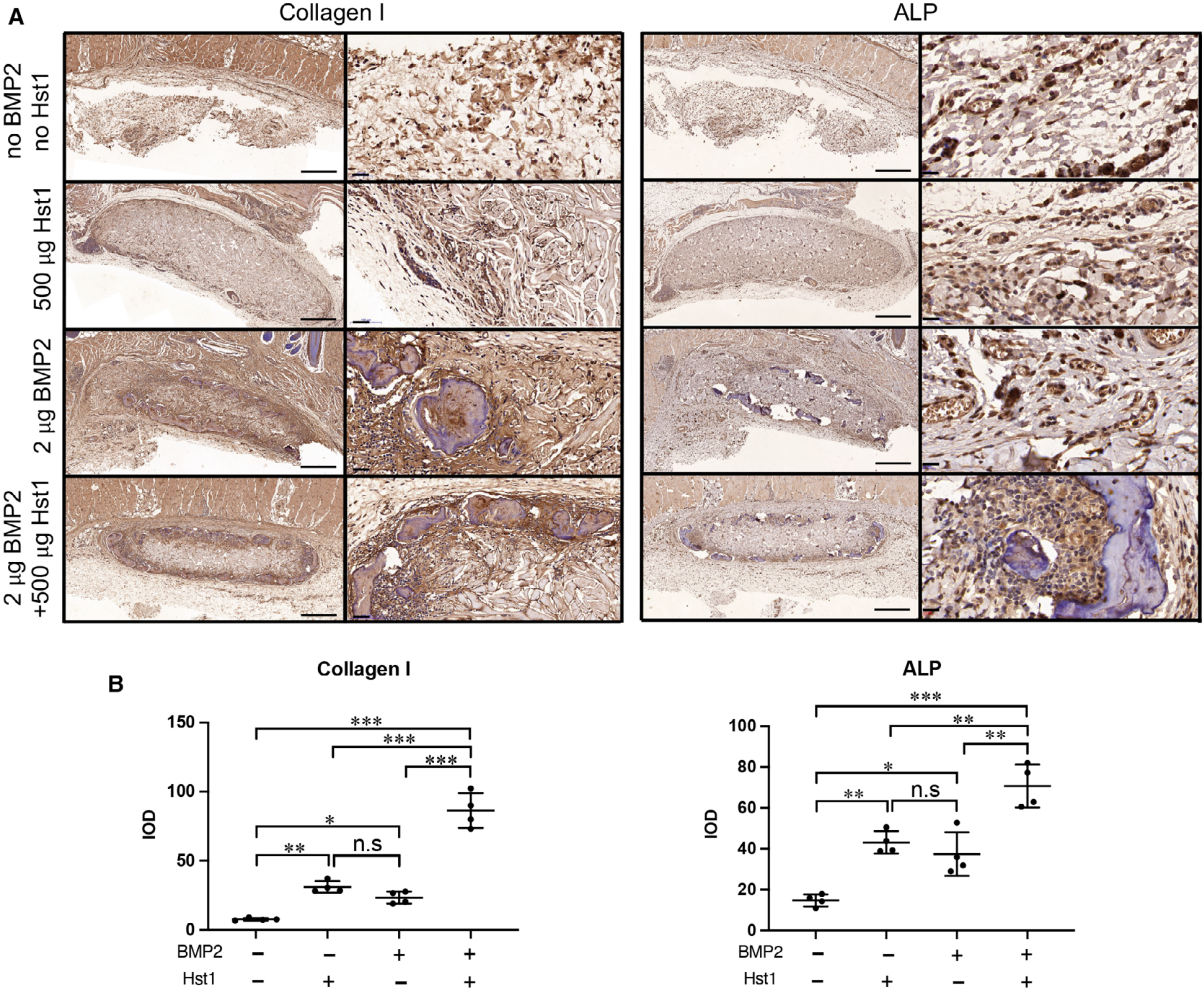
(A, B) Immunohistochemical micrographs (A) and quantitative analyses (B) of osteogenic markers collagen I and ALP were performed on ACS in four groups: control group (no Hst1, no BMP2), 500 μg Hst1 per sample, 2 μg BMP2 per sample and 2 μg BMP2 per sample + 500 μg Hst1 per sample. Scale bars: 500 μm in lower‐magnification images; 20 μm in higher‐magnification images. Integrated optic density (IOD) was calculated. Samples were retrieved after an 18‐day subcutaneous implantation. Data were analyzed by two‐way ANOVA with Bonferroni’s test for multiple comparisons. Data are presented as mean ± SD; *n* = 4 per group. Asterisks denote the level of statistical significance: *P < 0.05, **P < 0.01, ***P < 0.001. n.s., not significant.

To evaluate whether Hst1 could enhance ectopic bone formation, we assessed osteogenic markers Runx 2 (Fig. 3A), collagen I and ALP (Fig. 4A) by IHC. The addition of either 500 μg Hst1 or 2 μg BMP2 could significantly enhance the positive cell number of Runx2 to 18.0 ± 2.9 (*P* < 0.001) and 36.2 ± 9.9 (*P* < 0.001), respectively, in comparison with the control group (5.5 ± 1.3; Fig. 3B). The combination of 500 μg Hst1 and 2 μg BMP2 further enhanced positive cell number of Runx2 to 71.0 ± 11.3 (Fig. 3B). Similarly, the coadministration of Hst1 and BMP2 was associated with significantly higher expression of collagen I and ALP (86.4 ± 20.5 and 65.7 ± 17.7, respectively; *P* < 0.001) than the control (7.7 ± 0.9 and 14.7 ± 2.9, respectively) and BMP2 alone groups (23.4 ± 4.3 and 34.8 ± 13.2, respectively; Fig. 4B) in higher‐magnification images.

### IHC analysis of angiogenic markers

**Fig. 5:**
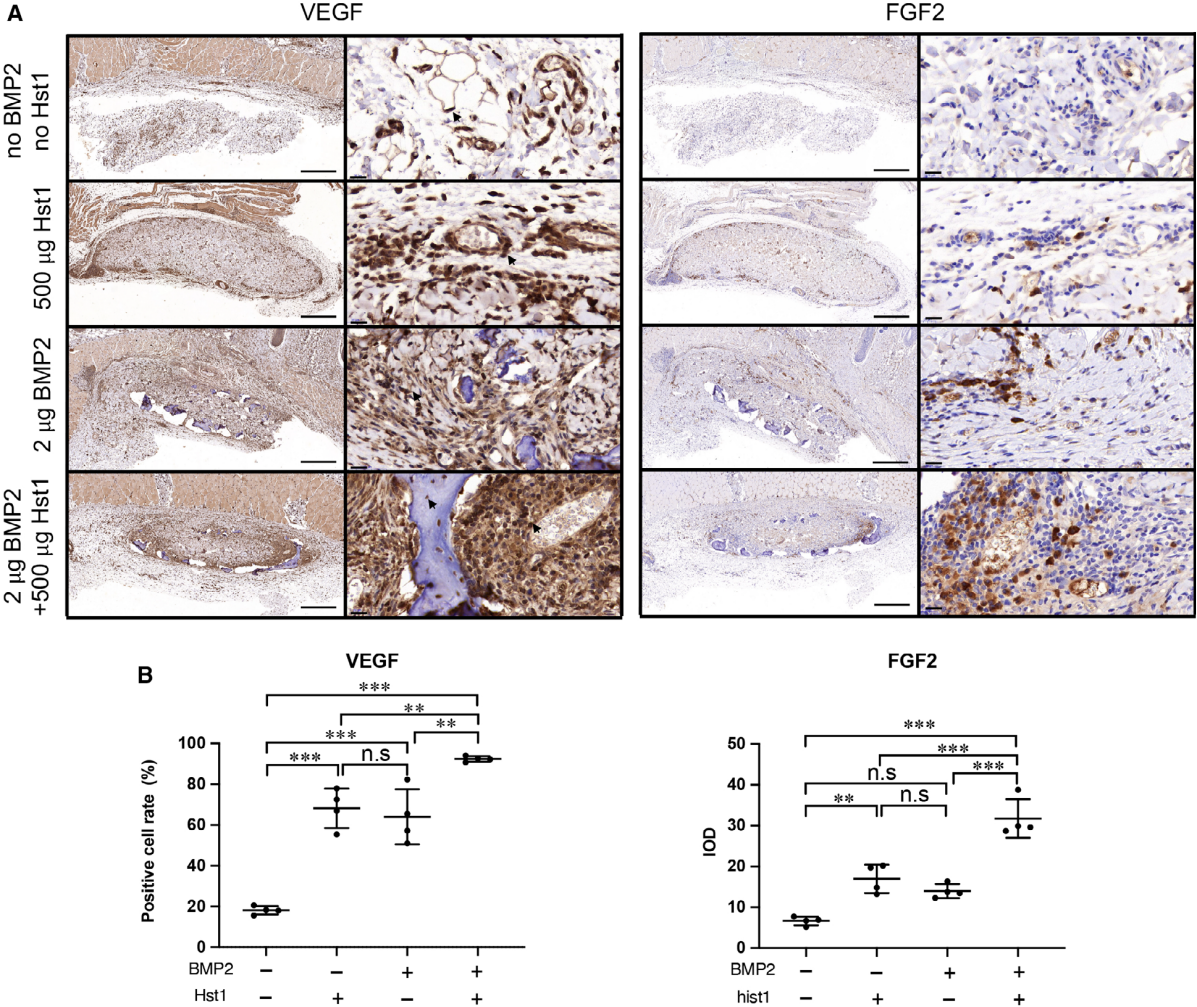
(A, B) Immunohistochemical micrographs (A) and quantitative analyses (B) of angiogenic markers VEGF and FGF2 on ACS were performed in four groups: control group (no Hst1, no BMP2), 500 μg Hst1 per sample, 2 μg BMP2 per sample and 2 μg BMP2 per sample + 500 μg Hst1 per sample. Cells with positive reaction were indicated by black arrows. Scale bars: 500 μm in lower‐magnification images; 20 μm in higher‐magnification images. The positive cell number within unit area (350.26 × 180.77 μm^2^) was quantified. Integrated optic density (IOD) was calculated. Data were analyzed by two‐way ANOVA with Bonferroni’s test for multiple comparisons. Samples were retrieved after an 18‐day subcutaneous implantation. Data are presented as mean ± SD; *n* = 4 per group. Asterisks denote the level of statistical significance: **P < 0.01, ***P < 0.001.

**Fig. 6:**
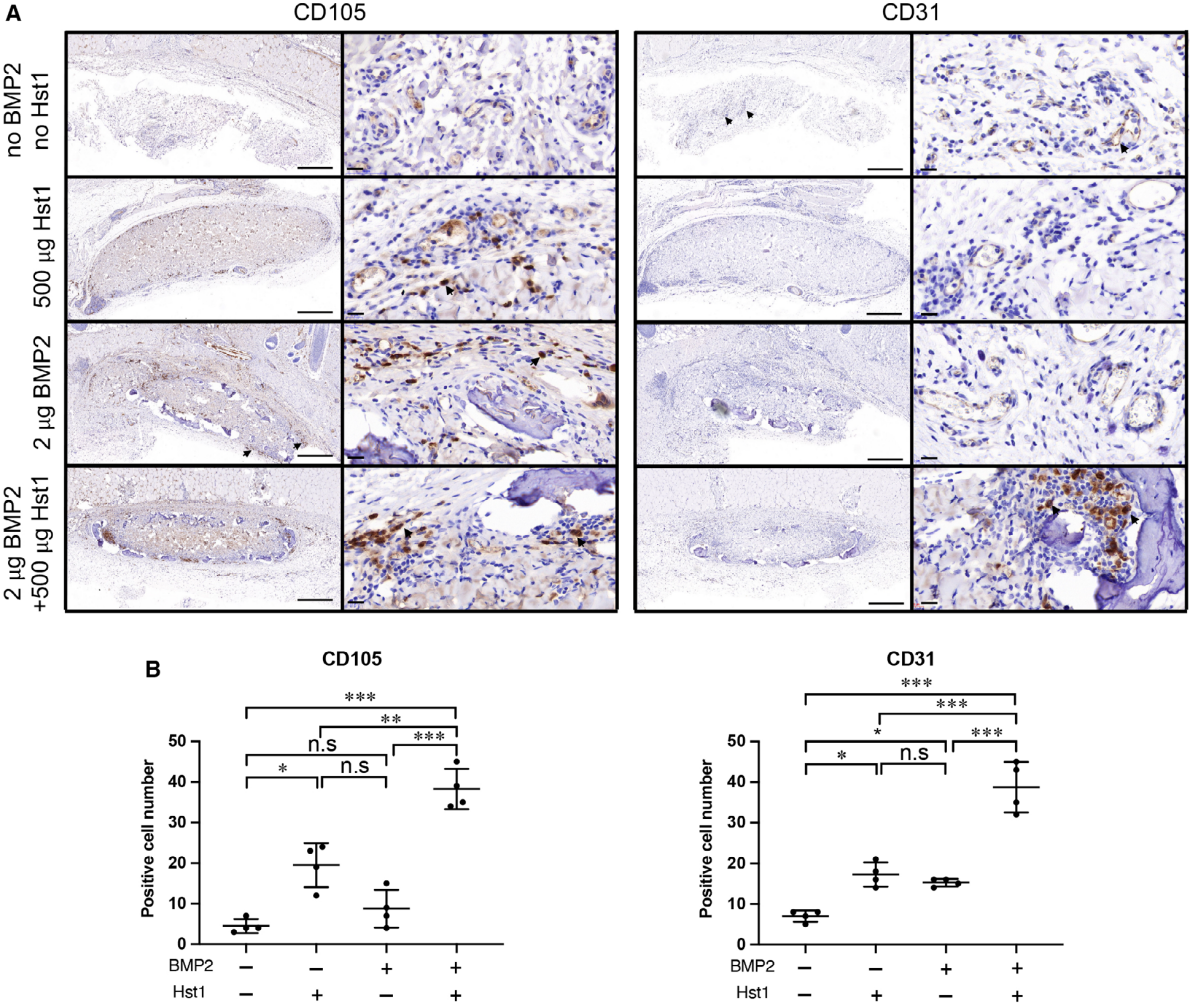
(A, B) Immunohistochemical micrographs (A) and quantitative analyses (B) of angiogenic markers CD105 and CD31 were performed on ACS in four groups: control group (no Hst1, no BMP2), 500 μg Hst1 per sample, 2 μg BMP2 per sample and 2 μg BMP2 per sample + 500 μg Hst1 per sample. Cells with positive reaction were indicated by black arrows. Scale bars: 500 μm in lower‐magnification images; 20 μm in higher‐magnification images. Positive cell number within unit area (350.26 × 180.77 μm^2^) was quantified. Data were analyzed by two‐way ANOVA with Bonferroni’s test for multiple comparisons. Samples were retrieved after an 18‐day subcutaneous implantation. Data are presented as mean ± SD; *n* = 4 per group. Asterisks denote the level of statistical significance: *P < 0.05, **P < 0.01, ***P < 0.001.

To explore the angiogenic effect to the additional Hst1, the expression levels of angiogenic markers, such as VEGF, FGF2 (Fig. 5A), CD105 and CD31 (Fig. 6A), were examined with IHC analysis, respectively. BMP2, Hst1 or their combination could significantly enhance VEGF by almost 3.0‐fold (*P* < 0.001; Fig. 5B). For FGF2, CD105 and CD31, BMP2 or Hst1 alone could significantly enhance these parameters. Furthermore, the coadministration of BMP2 and Hst1 synergistically enhanced these parameters (Figs 5B and 6B).

## Discussion

Sufficient BV is highly important for musculoskeletal functions and patient aesthetics. BMP2 is widely used in clinic for bone regeneration, and continuous efforts have been taken to enhance the osteoinductive efficacy of BMP2. In this study, we, for the first time, showed that human salivary Hst1 could significantly promote BMP2‐induced bone regeneration and related osteogenic markers. Furthermore, we showed that Hst1 and BMP2 could significantly promote the expression of angiogenic growth factors and new blood vessels formation. Our data suggested a promising application potential of Hst1 in promoting the repair of large‐volume bone defects.

Osteoinductivity is a feature of inducing the differentiation of MSCs down an osteoblastic lineage [26]. In this study, we adopted the classic ectopic (subcutaneous) bone induction model for indicating osteoinductivity of bioactive agents [27]. Because we did not use any exogenous stem cells or osteoblasts, and there was no existing bone tissue in the surrounding tissue, endogenous MSCs facilitated osteogenesis and angiogenesis. The MSCs may be recruited from: (a) surrounding connective tissues[27] and the (b) bloodstream [28]. In our study, new bone formation was observed only in the BMP2‐containing groups, which indicated that BMP2 possessed osteoinductivity, whereas Hst1 did not.

Hst1 belongs to a large family of histidine‐rich peptides that constitute a very important defense mechanism in the oral cavity. Apart from antimicrobial effects [29], Hst1 bears a potent capacity to accelerate wound healing [30]. Recently, Hst1 has been shown to be effective also for osteogenic cells [31]. It has been found that Hst1 promotes the adhesion and spreading of osteogenic cells *in vitro* [18] even in the cytotoxic and antimigratory conditions created by bisphosphonate [32]. Furthermore, Hst1 was also recently shown to be potent in inducing angiogenesis [22]. Consequently, Hst1 bears an application potential in promoting bone healing.

Most of the pharmacological concentrations of Hst1 have been obtained from *in vitro* cell studies. An *in vitro* wound healing study showed that 10 μm (about 50 μg·mL^−1^) was the optimal concentration [33]. An *in vivo* study showed that one drop of Hst1 at 0.1, 1 and 10 μg·mL^−1^ three times per day is equally effective in promoting corneal epithelial wound healing in rabbits. Albeit, it was still very difficult to determine the dosage of Hst1 in the current model because: (a) Hst1 was applied only once before implantation, and (b) blood flushing and enzymatic degradation will significantly reduce the available amount of Hst1 for its biological effect. We used a thumb principle for its dosage estimation: the most effective concentration of BMP2 to induce *in vitro* osteoblastogenesis is about 0.05–0.2 μg·mL^−1^ [34], and we used 2 μg BMP2 per sample *in vivo* as we previously described [10]. The *in vitro* concentration of Hst1 is 5–50 μg·mL^−1^ [20], and *in vivo* dosage can be 20–500 μg. Therefore, we determined to select Hst1 at 50, 100 and 500 μg per sample as a testing concentration. Further dose‐dependent study may also be performed to identify the higher dosage, such as 1, 2 and 5 mg.

The quality and quantity of newly formed bone can be evaluated by assessing both volumetric properties and geometric parameters [35]. For this purpose, microCT has been considered a gold standard instrument to evaluate both mineral density and 3D microarchitectures [36]. Our screening experiments revealed that Hst1 could dose‐dependently enhance BMP2‐induced new bone formation. We further evaluated bone strength and fracture resistance by assessing trabecular microstructures, such as Tb.Th., Tb.N., Tb.Sp., Tb.Pf. and B.S. [37]. At 18 days postimplantation, the presence of Hst1 did not significantly influence the Tb.Th. of BMP2‐induced new bone, but dose‐dependently promoted Tb.N. Hst1 at 500 μg per sample almost doubled the value in comparison with the BMP2 alone group. The significant increase of B.V. and Tb.N. contributed to the increase of B.S. Consistently, Hst1 also dose‐dependently reduced Tb.Sp. Tb.Pf. is a parameter to describe the relation of convex to concave surfaces. More concave surfaces represent a well‐connected spongy lattice [38]. All of the groups with Hst1 were associated with higher Tb.Pf., and Hst1 at 50 μg per sample increased Tb.Pf. by about 1.5‐fold in comparison with the 2 μg BMP2‐treated group (Fig. 1B). All of these results indicated that Hst1 could not only promote the BMP2‐induced osteogenesis but also improve the microstructures of the new bone. BMP2‐induced bone regeneration can be accelerated via many mechanisms, such as: (a) directly enhancing BMP2 signaling and (b) improving osteogenic microenvironments. Hitherto, there are few reports on signaling mechanisms of Hst1. Our recent study showed that ERK and p38 MAPK signaling was involved in Hst1’s promoting effect on the spreading of MC3T3‐E1 preosteoblasts (a mouse cell line) (data not shown), and extracellular signal‐regulated kinase (ERK) and p38 MAPK signaling are also actively involved in the BMP2‐induced osteogenesis [39]. Consequently, ERK and p38 MAPK signaling might mediate the promoting effect of Hst1 on BMP2‐induced osteogenesis.

Light micrographs showed that new bone formation mainly occurred at the periphery of the ACS constructs in the group of BMP2 alone. This distribution pattern of new bone was also observed in our recent study [10]. In contrast, the addition of Hst1, particularly at 500 μg per sample, resulted in more mineralization in the central region of ACSs (Fig. 2A). These findings indicated that Hst1 improves osteogenesis not only in quality and quantify but also in the distribution pattern of new bone within scaffolds. One potential mechanism accounting for this phenomenon is the promoting effects of Hst1 on cell adhesion and migration [30], thereby enhancing the invasion of cell and tissue into ACS constructs.

Hst1 may also benefit BMP2‐induced osteogenesis through enhancing angiogenesis, a prerequisite for bone regeneration [40]. Angiogenesis is composed of several cellular events, such as proliferation, migration and reorganization, into functional vascular structures of endothelial cells [41]. Several signals have been shown to initiate and sustain these responses, which include VEGF and FGF2 [41, 42]. Newly formed vessels can be symbolized by biomarkers, such as CD105 and CD31. CD105 (Endoglin) is an accessory receptor for transforming growth factor‐β, and its expression is up‐regulated in actively proliferating endothelial cells [43]. CD105 is recognized as an appropriate marker for neovascularization [44]. CD31, also named platelet/endothelial cell adhesion molecule‐1, is responsible for maintaining and restoring the vascular permeability barrier following disruption of the endothelial cell junction [45]. CD31 marks both neoformed vessels and normal preexistent [51]. CD105 is to be more specifically for the endothelial cells of neoformed vessels. Its expression increased in the same time with the neoangiogenic progression [46, 47]. BMP2 is unable to increase the rate of bone healing when there is inadequate vascularization in certain critical‑sized bone defects [48]. Previous studies have shown that, following treatment with BMP2, 25% of non‑union fractures required a secondary bone graft procedure, because of the lack of adequately vascularized tissue [49]. BMP2 can enhance both formation and neovascularization in critical size bone defects [50], which is consistent with our result that BMP2 alone significantly enhanced osteogenesis and angiogenesis. On the other hand, BMP2 does not directly stimulate angiogenesis *in vitro* [50]. In contrast, conditioned medium derived from both mesenchymal progenitor cells and osteoblasts can induce endothelial cell migration *in vitro*, suggesting a paracrine mechanism of BMP2 action [50]. Hst1 was recently shown to promote endothelial cell adhesion, migration and angiogenesis in both *in vitro* cell models and *in vivo* chick chorioallantoic membrane (CAM) assay [22]. Such effects of Hst1 are mediated through the activation of the Ras and Rab interactor 2/Rab5/Rac1 signaling axis [22]. In this study using a classical bone induction model, we, for the first time, provided evidence that Hst1 alone significantly enhanced the expression of a series of angiogenic markers, such as VEGF, FGF2, CD105 and CD31, which might partially account for the significant enhancement of new bone formation. Albeit, the present result was not sufficient to show the causal relationship between increased angiogenesis and enhanced new bone formation. Further studies are highly needed to investigate the underlying mechanisms accounting for the promoting effect of Hst1 on bone formation.

## Conclusions

Our results showed that the coadministration of Hst1 could significantly enhance BMP2‐induced osteogenesis and angiogenesis, which suggested a promising application potential of Hst1 in the treatment of large‐volume bone defects.

## Conflict of interest

The authors declare no conflict of interest.

## Author contributions

GW and PS conceived and designed the project. PS, YL and CS conducted the experiments. JF analyzed and interpreted the data. AS and CS wrote the manuscript. GW and JF supervised the work.
